# Poor-Law Reform

**Published:** 1905-08-26

**Authors:** 


### POOR-LAW REFORM.

### "INS AND OUTS."

Poor-law authorities are again trying to discover some method of dealing with those paupers who are known as "ins and outs." These people cause an amount of trouble quite disproportionate to the relief they receive. All the formalities of obtaining admission, all the ceremonies on entering, are required for men and women who perhaps want to leave again within a week. If they have children, these may be sent to the union schools—often situated at a considerable distance from the workhouse, and escorts have to be provided to take them there, and to bring them back when their parents resolve to go out. The right to indoor relief is abused by such people, whose proper resting-place is the casual ward, even though they may have acquired a settlement in the parish whose workhouse they periodically frequent. Some guardians put persistent "ins and outs" on the footing of casuals, while others, adopting an opposite policy, insist on seven days' notice of the paupers' intention to leave, and detain them for at least 168 hours after they return. The knowledge that if they go out, a bath awaits them on their return is said also to have a deterrent effect. Perhaps it might be possible to have other forms of probationary discipline, to be applied only to those whose persistent coming and going show that they are, or are likely to become, regular "ins and outs." The children of these people are much to be pitied, and are receiving just the sort of up-bringing which will tend to make them paupers themselves when they grow up. In the workhouse or the union school they have good food, clothing which is clean and whole, and a comfortable bed. These immediate advantages they can appreciate, and they are removed to the old life of dirt, starvation, wandering, and often blows, before the discipline and education by which they might profit have had time to do them good. They must remember the workhouse only as a haven of refuge, to which they will only too readily return. The remedy would be, of course, the adoption by the guardians of the children of "ins and outs," but to do this is simply to put a premium on parental carelessness and cruelty. Sometimes such a step is absolutely necessary, but one would hesitate to recommend adoption on a wholesale scale, which would put a heavy burden on the ratepayers, and make the path of the parents yet easier and more irresponsible. Another class of paupers seem also to need severer treatment than they at present receive. These are the able-bodied inmates who make the workhouse their permanent home, but go out now and then simply for a holiday. They take their discharge on the eve of Bank holidays, jus before the Derby, the Boat-race, or any local festival when they are likely to pick up from casual charity a sufficiency o food and drink, and coppers enough to pay for a lodging. They can thus pass a week-end or more, and when the effervescence of dole is past they lounge back to the workhouse to drowse and shirk until the next holiday comes round. The present practice of workhouse officials is to put no indignity on inmates going out for a day's leave. They are not compelled to wear uniform of any kind, and all that is demanded of them is that they return sober and at a proper hour, though when they fail in these respects their leave is stopped for some time. But this is another matter from people taking a formal discharge from the workhouse, as if they were going to make a new start in life, when they know, and all the officials know, that they have gone away only for a few days' holiday, and will be back in a week or so to the workhouse, their only permanent abode.

